# Striking a Balance—Cellular and Molecular Drivers of Memory T Cell Development and Responses to Chronic Stimulation

**DOI:** 10.3389/fimmu.2019.01595

**Published:** 2019-07-17

**Authors:** Jennifer L. Hope, Christopher J. Stairiker, Eun-Ah Bae, Dennis C. Otero, Linda M. Bradley

**Affiliations:** Tumor Microenvironment and Cancer Immunology Program, Sanford Burnham Prebys Medical Discovery Institute, La Jolla, CA, United States

**Keywords:** T cell memory, cancer, chronic infection, CD4 T cells, CD8 T cells

## Abstract

Effective adaptive immune responses are characterized by stages of development and maturation of T and B cell populations that respond to disturbances in the host homeostasis in cases of both infections and cancer. For the T cell compartment, this begins with recognition of specific peptides by naïve, antigen-inexperienced T cells that results in their activation, proliferation, and differentiation, which generates an effector population that clears the antigen. Loss of stimulation eventually returns the host to a homeostatic state, with a heterogeneous memory T cell population that persists in the absence of antigen and is primed for rapid responses to a repeat antigen exposure. However, in chronic infections and cancers, continued antigen persistence impedes a successful adaptive immune response and the formation of a stereotypical memory population of T cells is compromised. With repeated antigen stimulation, responding T cells proceed down an altered path of differentiation that allows for antigen persistence, but much less is known regarding the heterogeneity of these cells and the extent to which they can become “memory-like,” with a capacity for self-renewal and recall responses that are characteristic of *bona fide* memory cells. This review focuses on the differentiation of CD4^+^ and CD8^+^ T cells in the context of chronic antigen stimulation, highlighting the central observations in both human and mouse studies regarding the differentiation of memory or “memory-like” T cells. The importance of both the cellular and molecular drivers of memory T cell development are emphasized to better understand the consequences of persisting antigen on T cell fates. Integrating what is known and is common across model systems and patients can instruct future studies aimed at further understanding T cell differentiation and development, with the goal of developing novel methods to direct T cells toward the generation of effective memory populations.

## Introduction

**Figure 1:**
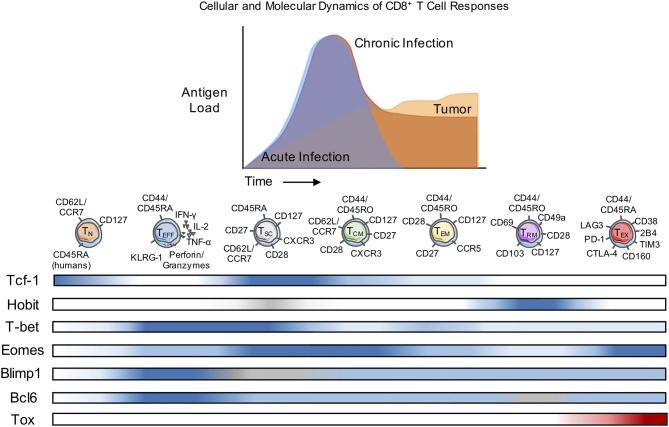
Cellular and Molecular Dynamics of CD8^+^ T Cell Responses. Naïve CD8^+^ T cells (T_N_) differentiate into diverse subsets with unique patterns of transcription factor (TF) expression and corresponding cell surface markers. The diversity of subset differentiation is highly influenced by the antigen or pathogen load in mice and patients. During acute infection, T_N_ cells give rise to polyfunctional, highly-proliferative effector (T_EFF_) CD8^+^ T cells that clear intracellular pathogens. Following contraction of T_EFF_ after antigen clearance, memory (T_CM_, T_EM_, and T_RM_) CD8^+^ T cells persist and rapidly respond upon re-infection. During chronic infection or in response to tumors, T_EFF_ also arise from Naïve, but often fail to effectively clear the infection or tumor and in response to persistent antigen can develop into T_EX_ with reduced function. In mice and patients, T_SC_ have been identified as a population of CD8^+^ T cells that respond to checkpoint blockade therapy. The development of classically-defined T_CM_, T_EM_, and T_RM_ during chronic infection or cancer remains contested and the differentiation of “memory-like” T cell populations is discussed within this review. Blue, level of expression of TF; white, no expression; gray, expression unknown; red, characteristic expression in T_EX_.

T cells are essential for the adaptive immune system's responses to pathogens and tumors. They are vital for the clearance of host cells that become infected with viruses and intracellular bacteria as well as the elimination of tumor cells (1, 2). T cell memory is typically defined as a residual compartment of protective antigen-specific T cell that persists long after contraction of the effector pool and survives in the absence of antigen (3). It is an important distinction that antigenic withdrawal does not occur during chronic infections and cancer (Figure 1) despite prolonged survival of responding T cells. Therefore, further insights are required into the differentiation of T cells in these contexts and in settings where clearance of a once chronic antigen ultimately occurs. These include (1) identifying characteristic phenotypic markers and transcriptional profiles, (2) ascertaining the capacity for self-renewal, and (3) determining the ability for rapid re-activation and generation of polyfunctional responses (4, 5). The focus of this review is to highlight the known differences in memory CD4^+^ and CD8^+^ T cell development in the context of chronic pathogen infections or cancer progression as compared to acute infections in both mice and humans, with an emphasis on the cellular and molecular drivers of T cell memory development under these conditions.

During the primary T cell response to infection or tumors, the antigen-specific T cell pool becomes highly heterogeneous, forming different subpopulations of CD4^+^ and CD8^+^ T cells defined by surface marker expression, transcription factors, cytokine production, and cytotoxic or memory-forming potential (Figure 1). Ultimately, with antigen clearance, the large majority of antigen-specific T cells die and a smaller pool of memory T cells that retain the capacity to respond to re-challenge can persist, often indefinitely (6, 7). However, memory T cells are also highly diverse, with substantial differences described for a variety of infections, implying the importance of contextual cues such as the duration of antigen exposure as well as the tissue localization and distribution of infection. Much less is known about the differentiation of memory CD4^+^ T cells compared to CD8^+^ T cells, in part because of the ability of naïve CD4^+^ T cells to adopt different effector cell fates that are uniquely regulated and are elicited by different infections. However, studies of circulating memory CD4^+^ (and CD8^+^) T cells in humans were the first to define effector memory T cells (T_EM_: CD45RA^+^CD127^+^CD62L^−^/CCR7^−^), and central memory cells (T_CM_: CD45RA^+^CD127^+^CD62L^+^/CCR7^+^) that primarily differ with respect to the circulation through secondary lymphoid organs and the capacity for self-renewal (T_CM_ > T_EM_) (8). In contrast to the extensive literature on the development of T cell memory to infections, much less is known about the characteristics of memory T cells that develop in responses to tumors.

There is considerable diversity in the fates of a naïve T cell and the mechanisms regulating the formation and promotion of heterogeneity in effector and memory T cell pools are of great interest, particularly in the context of vaccine development. Several models of memory T cell differentiation have been proposed and previously discussed elsewhere, but there is currently robust evidence for the “one cell, multiple fates” model (9, 10). In mice, fate-mapping and memory differentiation of CD8^+^ T cells that were assessed by Dirk et al. by performing adoptive transfer of single naïve OT-I TCR transgenic CD8^+^ T cells into recipient mice, followed by infection with OVA-expressing bacterium *Listeria monocytogenes* (Lm-OVA) (11, 12). These studies conclusively demonstrated that a naïve T cell could subsequently differentiate into both effector and memory T cells. Another study from Schumacher et al also assessed memory CD8^+^ T cell differentiation by fate-mapping analysis of adoptively transferred T cells, but used DNA-barcoded, transduced thymocytes from OT-I mice that were injected intra-thymically into young recipients, followed by infection with Lm-OVA (10). This study showed that a single antigen-specific naive CD8^+^ T cell gave rise to daughter cells with multiple phenotypes, including T_CM_ and T_EM_ subsets. Furthermore, the progeny of a single naïve CD8^+^ T cell could efficiently seed the secondary lymphoid organs (bone marrow, blood, spleen, and lymph nodes) and were not restricted to a particular anatomical location. Importantly, barcoded memory CD8^+^ T cells that were transferred into tertiary hosts maintained barcode diversity upon re-challenge, indicating the potential for all clones to respond. The fundamental question of whether effector T cells can give rise to memory cells was also demonstrated by fate mapping studies in which effector CD8^+^ T cells, identified by acquisition of the cytotoxic protein granzyme B, were shown to form memory (13). A more recent study demonstrated the ability of effector CD8^+^ T cells to “de-differentiate” into memory T cells by epigenetic remodeling associated with alterations in the DNA methylation programs of the cells (14). Together, these groups and others have demonstrated that indeed one naïve CD8^+^ T cell has the potential to give rise to daughter cells with differing phenotypes and fates, and that effector differentiation does not preclude memory development. However, to our knowledge such comprehensive studies addressing CD4^+^ T cell memory development have yet to be published and is a significant gap in understanding overall T cell memory differentiation.

Signals determining memory T cell generation remain incompletely understood. It is evident that antigen availability and timing of entry into a response are important determinants for memory formation. In general, weaker TCR signals are thought to favor memory T cell development, which can be influenced by TCR affinity, tissue localization with respect to antigen distribution, or by progressive antigen clearance (15). For CD8^+^ T cells, there is evidence that unique TCR signaling and organization of the TCR signaling complex that engages NF-kB signaling dictates memory development (16). These findings along with observations that CD8^+^ memory T cell precursors can be distinguished early in responses to acute infections in some models (e.g., LCMV Armstrong and *L. monocytogenes*) support the concept that early events are critical for memory development. Other external signals such as from cytokines during the effector phase also contribute to memory T cell differentiation. For example, type I interferon (IFN) or IL-2 signaling is key to the sustained survival of CD8^+^, and CD4^+^ T cells, respectively, during memory formation during the primary response (17, 18). Signaling through CD28 is required for the re-activation of memory CD8^+^ T cells and optimal recall responses of memory CD4^+^ T cells (7, 19). Co-stimulation of T cells such as through CD28 enhances their survival and effector function by increasing expression of the anti-apoptosis regulator BCL-_XL_, as well as by inducing T cell expansion, by the production of IL-2 (20, 21). Cytokines that include type I IFNs and IL-12 induce changes in the transcription factors T-box expressed in T cells (T-bet) and Eomesodermin (Eomes) (22, 23), which play important roles in regulating effector and memory T cell differentiation (24, 25) as summarized below. CD4^+^ and CD8^+^ T cells also influence each other during memory T cell development. Although CD4^+^ T cells are dispensable for the generation of primary effector CD8^+^ T cell responses to some infections, CD27 on CD8^+^ T cells interacting with CD70 on APCs primed by CD4^+^ T cell “help” via CD40(APC)/CD40L(CD4^+^ T cell) activation is required for the generation of functional memory CD8^+^ T cells marked by reduced proliferative capacity during recall responses (26).

Following a primary adaptive immune response, distinct subsets of memory T cells are found within the lymphoid organs that include not only T_CM_ or T_EM_, but also more recently defined memory cells that become resident in the initial site of the primary infection or tumor (T_RM_). All three subsets play roles in protective memory responses, although T_RM_ are likely to provide a first line of defense against a tissue localized re-infection. The T_RM_ compartment was first characterized in the skin where these cells control reinfection with herpes simplex virus (HSV), and have since been identified as key mediators of immunity in the lung, such as in response to RSV and influenza; and in the gut after infection with Lm or LCMV (27–29). Although T_RM_ have been identified by phenotype in tumors, their functions are not yet established (30). The T_RM_ pool contains two subsets distinguished by CD103 expression (also known as integrin alpha E), a receptor for E-cadherin (31). Current studies are focusing on possible functional differences between the CD103^+^ and CD103^−^ subsets of T_RM_. Another recently-defined memory T cell subset is considered to have stem cell-like properties with respect to self-renewal, and has been designated stem cell memory T cells (T_SCM_). Unlike other memory CD8^+^ T cell subsets, these cells maintain a naïve-like phenotype yet have a high proliferative capacity (32). Surface marker expression remains one of the primary methods for the classification of these different memory T cell subpopulations in both humans and mice (Table 1), and several distinctions apply more narrowly to memory cells generated in responses to specific infections. The presence of many of phenotypic and functional distinctions of memory cells has been much less well-studied in anti-tumor responses. It is now evident that a spectrum of surface phenotypes can arise primarily because of contextual cues and that these may be fluid and insufficient to fully define memory T cell subsets. However, in combination with analyses of transcription factor expression, greater insights into distinct features of memory T cell fates emerge.

**Table 1:** Expression profiles of CD8^+^ T cell subsets.

	**T_**N**_**	**T_**Eff**_**	**T_**EMRA**_ (Hu)**	**T_**CM**_**	**T_**EM**_**	**T_**EX**_**	**T_**RM**_**	**T_**SC**_**
**SURFACE MARKERS**								
CD62L	+ (33)	– (34, 35)	– (36)	+ (34, 35)	– (34, 35)	– (34)	– (31)	+ (37)
CCR7	+ (38)	– (34)	– (36)	+ (34, 35, 38)	– (34, 38)	– (34)	± (38)	+ (37)
CD44	– (33)	+ (33)	N/A	+ (35)	+ (35)	+	+	(low) (37)
CD45RA (Hu)	+ (39)	+ (39)	+ (39)	– (39)	– (39)	–	–	– (37)
CD45RO (Hu)	– (39)	– (39)	– (39)	+ (35, 39)	+ (35, 39)	+	+	+ (37)
CD122 (IL-2R β-chain)	– (33)	+ (33)	N/A	+ (33, 35)	+ (33)	– (33)	– (33)	+ (37)
CD127 (IL-15R)	+ (40)	– (40)	+ (40)	+ (35, 40)	+ (35, 40)	+	± (41)	+ (37)
CD27	+ (40, 42)	– (40, 42, 43)	± (39, 40)	+ (40)	+ (40)	N/A	± (41)	+ (37)
CD28	+ (40, 42)	– (42)	– (39, 40)	+ (40)	+ (40)	N/A	± (41)	+ (37)
KLRG1	– (40, 43)	+ (35, 40, 43)	+ (40)	– (37, 40)	+ (37, 40)	N/A	N/A	– (37)
CXCR3	– (44)	± (44, 45)	N/A	+ (44, 45)	^−^(44)	N/A	(low) (46)	+ (37)
PD-1 (CD279)	– (47)	+ (47)	± (40)	± (40, 47)	± (40, 47)	+ (47, 48)	± (41)	+ (49)
CTLA-4 (CD152)	– (50)	+ (50)	N/A	(low) (50)	(low) (50)	+ (35, 48)	N/A	N/A
LAG-3 (CD233)	– (50)	N/A	N/A	+	+	+ (35, 48)	N/A	N/A
TIM-3 (CD366)	– (50)	(low) (50)	N/A	N/A	N/A	+ (35, 48)	N/A	N/A
2B4 (CD244)	– (50)	– (51)	N/A	N/A	N/A	+ (48)	N/A	N/A
CD160	– (50)	– (51)	N/A	N/A	N/A	+ (48)	N/A	N/A
CD69	– (52)	+ (35)	N/A	– (38)	– (38)	+ (48)	+ (31, 38)	+ (49)
CD103	N/A	N/A	N/A	– (38)	– (38)	N/A	± (38)	N/A
Sca1	N/A	N/A	N/A	N/A	N/A	N/A	N/A	+ (37)
CXCR5	– (50)	N/A	N/A	N/A	N/A	– (49)	N/A	+ (49)
**CYTOKINES/CYTOTOXIC GRANULES**								
IL-2	– (53)	+ (48)	+ (36)	+ (51)	+ (51)	– (48)	+ (41)	+ (54)
IFNγ	– (50)	+ (48)	+ (36)	+ (51)	+ (51)	– (48)	+ (41)	+ (54)
TNFα	– (53)	+ (48)	N/A	+ (51)	+ (51)	– (48)	+ (41)	+ (54)
Perforin	– (53)	+ (48)	+ (55)	(low) (51)	– (51)	– (48)	N/A	(low) (54)
Granzyme B	– (50)	+ (48)	+ (36)	(low) (51)	+ (51)	– (48)	+ (41)	(low) (54)
**TRANSCRIPTION FACTORS**								
Tcf1	(high) (56)	– (57)	± (56)	(med) (35, 56)	(low) (56)	– (56)	– (58)	(high) (49)
Foxo1	(high) (59)	+ (59)	+ (59)	+ (59)	+ (59)	+ (59)	N/A	N/A
Runx3	+ (60)	+ (35, 60)	N/A	+ (60)	+ (60)	+ (61)	+ (62)	N/A
ID2	– (50)	+ (35, 50)	N/A	N/A	+ (63)	+ (50)	N/A	N/A
ID3	+ (50)	+ (50)	N/A	+ (35, 63)	N/A	+ (50)	N/A	N/A
Tbet	– (50)	(high) (35)	+ (64)	(low) (35)	(med) (35)	(low) (35)	(low) (63)	+ (37)
Eomes	– (50)	(med/hi) (35)	N/A	(high) (35)	(med) (35)	(high) (35)	(low) (63)	+ (37)
Blimp1	– (50)	(high) (35)	N/A	+ (50)	+ (35, 50)	+ (35, 50)	+ (63)	N/A
Bcl6	– (65)	+ (65)	N/A	+ (35)	+ (35)	+ (57)	N/A	+ (57)
IRF4	– (66)	+ (66)	N/A	(low) (50)	(low) (50)	(high) (50)	(low)	(low)
BATF	– (50)	(low) (50)	N/A	– (50)	– (50)	+ (35, 50)	N/A	N/A
Hobit	– (67)	– (67)	+ (68)	– (67)	– (67)	– (67)	+ (63, 67)	N/A
Tox	– (50)	– (50)	N/A	– (50)	– (50)	+ (50, 69–73)	– (69)	N/A

*      Denotes expression observed primarily in mice*.

*N/A Denotes expression either unknown or not discussed within this review*.

Transcription factors are well-recognized as key regulators of T cell fate determination. In both CD4^+^ and CD8^+^ T cells, opposing levels of transcription factor pairs can strongly correlate with the T cell memory subsets. Examples of these gradients of transcription factors are T-bet vs. Eomes, B lymphocyte-induced maturation protein-1 (Blimp-1) vs. B-cell lymphoma 6 (Bcl-6), and Inhibitor of DNA binding 2 (Id2) vs. Inhibitor of DNA binding 3 (Id3) (65, 74, 75). At the memory stage, Eomes is more highly expressed than T-bet. Similarly, while Blimp-1 is highly expressed in effector cells and reciprocally Bcl-6 expression increases in memory cells; a similar relationship has been observed between Id2 and Id3. There is also a supportive role for transcription factor expression during early differentiation in memory formation and maintenance, specifically the Forkhead box proteins O-class proteins (Foxo) Foxo1 and Foxo3 (59, 76). Understanding how these transcription factors interact with each other remains an active area of research; for example, Foxo1 is known to regulate other transcription factors such as by increasing Tcf-1, Eomes, and Bcl-2 expression, while repressing the levels of T-bet. Within memory T cell subsets, there are also unique expression patterns of transcription factors, several of which are outlined in Table 1 and highlighted in Figure 1. These transcription factors are altered in exhausted T cells (T_EX_), and interestingly, expression patterns of some transcription factors associated with memory T cell formation are shared with T_EX_ that persist during chronic infection or cancer suggesting that re-stimulation of more “memory-like” T cells could contribute to achieving a balance between terminal T cell differentiation and pathogen or tumor control and the extent of T cell exhaustion, which has been extensively reviewed elsewhere (77).

Changes in the stimulatory conditions encountered by effector T cells could also impact the development of memory T cells, as reduced inflammation caused by increasing antigen control could limit the extent of the effector T cell response, particularly in the infection or tumor the draining lymphoid tissues. Thus, conditions that are highly influenced by: (1) the cellular microenvironment, and (2) changes in the molecular regulation of the responding cells are likely to be key in determining whether T cells become terminally differentiated or “memory-like” and thus lead to a spectrum of functionally heterogeneous T cells. An important consideration when contrasting T cell differentiation and development in responses to chronic infection or tumors is the influence of a highly systemic inflammatory response observed in some chronic infections and model systems compared to the more localized microenvironment typically associated with the early stages of cancer. However, there is evidence in both humans and mice of memory or “memory-like” CD4^+^ and CD8^+^ T cell formation under conditions of repeated stimulation. The following sections will outline the effects that persistent pathogenic infections or tumors have on CD4^+^ and CD8^+^ memory or “memory-like” T cell development and responses, and the interplay between the two.

## T Cell Responses to Chronic Infection

### CD8^+^ Memory T Cell Development in Chronic Infection

The defining cellular environment of memory CD8^+^ T cells is a compilation of interactions with other cellular compartments and the localization of the cell (such as in circulation, lymphoid tissues, or non-lymphoid tissues). In both acute infections and upon re-challenge, secondary lymphoid organs such as the peripheral lymph nodes are important sites of naïve or memory CD8^+^ T cell activation during systemic viral infections such as LCMV. Here, dendritic cells (DC) that have captured and present LCMV antigens activate CD8^+^ T cells. Surrounding the interacting T cell-DC conjugates are fibroblastic reticular cells (FRC), which can either promote and accelerate T cell activation, or conversely can inhibit subsequent expansion within the lymph nodes via the production of nitric oxide (78). FRC also constitutively express PD-L1, the ligand for the T cell inhibitory receptor PD-1 (programmed cell death protein 1, CD279) (79). During chronic LCMV infection, PD-L1 expression on FRCs is elevated and the network that supports T cell migration is disrupted, leading to altered localization (80). These changes in the lymphoid tissue architecture are thought to promote T cell exhaustion and impede memory formation. Persistent viral infections, such as LCMV in mice and Human Immunodeficiency Virus-1 (HIV-1) and Hepatitis C Virus (HCV) in humans, can also result in the inhibition and loss of type 1 T helper (T_H_1) CD4^+^ T cell responses that play a major role in supporting memory CD8^+^ T cell development (81–83). During LCMV chronic infection, there is a progressive decline in virus-specific CD8^+^ T cells; however, reconstituting the T_H_1 CD4^+^ T cell compartment is sufficient to maintain the LCMV-specific CD8^+^ T cell population, providing greater evidence for a supportive role for CD4^+^ T cells in maintaining long-lasting CD8^+^ T cells that continue to undergo progressive differentiation (83).

Localization of virus-specific CD8^+^ T cells in other non-lymphoid sites such as the kidney, liver, and lungs during chronic LCMV infection has been previously described, with evidence that they too exhibit signs of functional exhaustion with decreased IFN-γ and TNF-α production upon *ex vivo* stimulation compared to conventional LCMV-specific memory CD8^+^ T cells (84). Similarly, in a chronic parasitic infection model of *Trypanosoma cruzi* in mice, muscle-resident CD8^+^ T cells have decreased effector function (85). The majority of long-lived CD8^+^ T cells in the lung, liver, and kidneys after chronic LCMV infection fail to express CD103; however, LCMV-specific intraepithelial CD8^+^ T cells found within the small intestine and lamina propria express both CD103 and CD69 (86), which establishes tissue localization via the G-protein-coupled receptor sphingosine-1-phosphate receptor (S1PR1) (87). However, whether these T_EX_ in non-lymphoid tissues share features with T_RM_ or provide a major role in maintaining chronic infection has not been studied.

While our understanding of CD8^+^ T cell differentiation and “memory-like” development during chronic infections has largely been derived from mouse model systems, several studies have focused on dissecting human virus-specific CD8^+^ T cell differentiation under persistent viral infections including HIV-1, HCV, Epstein Barr virus (EBV), and cytomegalovirus (CMV) through the use of Human Leukocyte Antigen class I (HLA-I) tetramers complexed with peptides of virus-derived CD8^+^ T cell-specific epitopes. Both CD27 and CD28 expression levels have been used to classify the differentiation state of CD8^+^ T cells and are regularly used in connection with CD45RA and CCR7 to distinguish effector and memory T cells. One study identified unique patterns of CD8^+^ T cell differentiation in the periphery based on the specific viral infection, finding a greater frequency of CD28^+^ virus-specific CD8^+^ T cells from HCV-infected patients compared to HIV, CMV, or EBV; conversely, the frequency of CD27^+^ virus-specific CD8^+^ T cells was lower in CMV (88). The expression of CD57, meanwhile, has been linked to both CD8^+^ T cell memory subsets (both T_CM_ and T_EM_) but also senescent or functionally exhausted CD8^+^ T cells, adding to the complexity of differentiating between “memory-like” vs. exhausted human CD8^+^ T cell subsets (89, 90). In some patients, infection with *Mycobacterium tuberculosis* (TB) can result in latent infection (LTBI) where the bacteria remain quiescent until re-activation. Unlike during active TB infection, the differentiation of CD8^+^ T cells in LTBI patients is highly skewed toward terminally differentiated effector memory cells (T_EMRA_) as opposed to the T_EM_ compartment (91). Together, these highlighted studies demonstrate how different infections, despite their chronicity or latency, can drive a highly heterogeneous memory CD8^+^ T cell population in patients.

An important consideration in defining T_EX_ is the co-expression of inhibitory receptors including PD-1, CTLA-4, LAG3, TIM3, 2B4, and CD160. Expression of a single inhibitory receptor is insufficient to define T_EX_, as some inhibitory receptors such as PD-1 are upregulated upon T cell activation and therefore can also serve as activation markers. A major distinction between exhausted and memory CD8^+^ T cells is the ability to produce cytokines such as IFN-γ, TNF-α, and IL-2 upon TCR stimulation. Terminally exhausted T cells, initially described in the chronic LCMV infection model in mice, demonstrate a marked reduction or inability to produce these cytokines upon re-stimulation, and it is this concurrent loss of polyfunctionality (the ability to produce multiple cytokines and mediate toxicity) and increasing inhibitory receptor co-expression that is crucial when defining T_EX_. Greater insight into T cell exhaustion including molecular and cellular drivers of exhaustion is thoroughly reviewed in McLane et al. (77). In contrast, re-stimulation of memory CD8^+^ T cells results in a high level of cytokine production that is associated with low co-expression of inhibitory receptors. Insight into T cell responses during chronic infection was provided by the observation that antigen-load plays a crucial role in the development of an exhausted CD8^+^ T cell phenotype, as decreasing the abundance solely of GP33 (an LCMV-derived epitope recognized by CD8^+^ T cells) on the virus while maintaining viral loads and other LCMV-derived epitopes resulted in reduced expression of PD-1 and elevated dual-production of IFN-γ and TNF-α by P14 (GP33-specific TCR transgenic) CD8^+^ T cells (92). In support of “memory-like” CD8^+^ T cell development during this infection is the finding that virus-specific T cells transferred into naïve mice 4 weeks after initial infection with LCMV Cl13 were able to expand and control viral titers when recipient mice were infected with the acute virus LCMV Armstrong strain, despite retaining high PD-1 expression levels and reduced (but not absent) IFN-γ and TNF-α production (93). Further, these antigen-specific CD8^+^ T cells were maintained in the absence of antigen or infection, a foundational hallmark of memory T cells, through signaling from the homeostatic cytokines, IL-7 and IL-15, via their receptors CD127 (IL-7R) and CD122 (IL-15R). This is in contrast to an earlier finding which demonstrated that memory CD8^+^ T cells isolated from the very late time-point of 120 days post-infection fail to persist or respond to LCMV after transfer into naïve host mice (94). Moreover, with chronic LCMV infection, long-lived virus-specific CD8^+^ T cells during chronic LCMV infection show decreased expression of both CD127 and CD122 (95, 96). It is likely that fewer CD8^+^ T cells present in chronically infected hosts at day 28 post-infection have yet to be driven to terminal differentiation as compared to the very late time-point of 120 days.

A hallmark of memory CD8^+^ T cells is their capacity to proliferate upon TCR engagement, whereas T_EX_ are ultimately driven toward apoptosis. Several studies have evaluated how chronic infection affects CD8^+^ T cell differentiation and impacts memory or “memory-like” T cell populations under these conditions. The discovery that checkpoint inhibitor blockade, notably through the use of anti-PD-1 and anti-CTLA-4 antibodies, reinvigorates exhausted T cells was a landmark finding that ultimately changed the landscape of cancer therapy. The important groundbreaking work by the pioneering studies on CTLA-4 and PD-1 by James Allison and Tasuku Honjo, respectively, was recently recognized by their receipt of the Nobel Prize in Medicine in 2018. Blockade of the PD-1/PD-L1 pathway was also found to abrogate T cell exhaustion when therapeutically administered to mice persistently infected with chronic LCMV (97, 98). While early interpretations of these data suggested the reversal of T cell exhaustion, more recent studies have identified a unique “memory-like” subset of exhausted T cells (T_SC_) that is responsible for the T cell response with PD-1 blockade therapy. One study has demonstrated that CD28 signaling is a cell-intrinsic requirement for LCMV-specific cells to proliferate in response to anti-PD-1 treatment (99). Further examination of PD-1^+^ cells in LCMV Cl13-infected mice identified CXCR5 expression as a distinguishing marker of PD-1 blockade responsiveness (49). Transcriptional profiling of CXCR5^+^ cells identified the expression of several Wnt signaling genes associated with self-renewal and hematopoietic stem cell maintenance (49). Importantly, this subset was also found to have high levels of Id3 over Id2, and high Eomes over T-bet—transcription factor profiles characteristic of memory precursor and memory CD8^+^ T cells (49). T_RM_ also have unique transcriptional signatures from other memory T cell compartments, such as the expression patterns of transcription factors Blimp-1 and Hobit (Zfp683, “homolog of Blimp-1 in T cells”) which are co-expressed in T_RM_, with simultaneous low expression of Eomes and T-bet (86). In contrast, ZBTB32 (zinc finger and BTB domain containing 32) is another transcription factor co-expressed with Blimp-1 and limits CD8^+^ T cell memory development during both acute and chronic viral infections (100).

Further support for “memory-like” CD8^+^ T cell development during LCMV Cl13 infection was the identification of a role for the transcription factor T cell factor-1 (Tcf-1, encoded by the gene *Tcf7*) in a subpopulation of virus-specific CD8^+^ T cells. Previously described as a transcription factor co-activated by β-catenin downstream of canonical Wnt signaling, Tcf-1 was found to play a role in memory CD8^+^ T cell generation and function (101, 102). The use of Tcf-1 reporter mice identified that Tcf-1 expression in CD8^+^ T cells was associated with the maintenance and re-expansion of virus-specific CD8^+^ T cells in LCMV Cl13 infected mice. RNA-seq analysis of Tcf-1-expressing cells showed transcriptional characteristics that were shared with both memory and exhausted CD8^+^ T cells, but unique from effector T cells (103). In support of their characterization as a “memory-like” T cell compartment, Tcf-1-expressing CD8^+^ T cells show low levels of KLRG1, CX3CR1, T-bet, Blimp1, and granzymes while expressing high levels of IL-7R, CD62L, CCR7, Id3, and Bcl-6. However, Tcf-1-expressing cells share PD-1, LAG3, and c-Maf expression levels on par with exhausted T cells, supporting the concept that these cells are unique from archetypal memory cells. In humans, similar characteristics in Tcf-1-expressing cells including the ability to expand upon re-challenge stimulation were described in HCV-specific T cells (of which 20–60% were Tcf-1^+^), demonstrating that this is not a LCMV-specific phenomenon (103). Indeed, further studies involving HCV-infected patients attribute the heterogeneity of memory CD8^+^ T cells to differing levels of Tcf-1 expression (56). By assessing the graded expression levels of Tcf-1, a recent study found a reciprocal relationship between T-bet and Tcf-1, while Eomes expression was highest within the Tcf-1-intermediate compartment (56). While these studies have led to a greater understanding of CD8^+^ T cell biology, most importantly they led to an important connection between Tcf-1 expression and CD8^+^ T cell responsiveness to PD-1-targeted checkpoint blockade therapy in cancer, which is discussed below.

The vast heterogeneity of differentiated and “memory-like” CD8^+^ T cells that arise during persistent antigen exposure demonstrates the importance in understanding the cellular and molecular drivers of protective immunity. Importantly, we must better understand the conditions that give rise to “memory-like” exhausted T_SC_ CD8^+^ T cells, as these appear to be the cells most responsive to checkpoint blockade therapy and therefore less sensitive to terminal exhaustion. Such insights are needed to instruct the future development of new immunomodulatory checkpoint blockade therapies, establish whether a patient would be responsive to therapy, and enhance vaccination strategies.

### CD4^+^ Memory T Cell Development in Chronic Infection

The importance of CD4^+^ T cells during persistent infections is highlighted by models in which this immune cell compartment is depleted. During chronic infection in humans and mice, decrease in helper CD4^+^ T cells or their functional capacity is associated with less pathogen control or the establishment of chronicity (104, 105). Further, the CD4^+^ T cell compartment plays a pivotal role by contributing to both the cellular and humoral arms of the immune response in chronic infection in both mice and humans (106). Despite the importance of the CD4^+^ T cell response during chronic infection, greater emphasis and research has focused on understanding their role in CD8^+^ T cell differentiation during chronic infection; much less is known about how chronic antigenic stimulation affects the differentiation and subsequent “memory-like” population of persisting CD4^+^ T cells in the context of persistent infection.

It is a well-defined phenomenon that CD8^+^ T cells become exhausted as a result of continuing antigenic exposure during chronic infections, as summarize above. Whether this is true for CD4^+^ T cells remains unclear, although the development of dysfunction clearly occurs. Using the LCMV models to compare acute vs. chronic viral infections, CD4^+^ T cells demonstrate a reduced characteristic T_H_1 cell cytokine profile, i.e., reduced production of IFN-γ, TNF-α, and IL-2 (107, 108). HCV infection is also known to cause an acute infection that can progress to chronicity if not controlled. In peripheral blood, broadly reactive CD4^+^ T cells were detected early during this infection but became undetectable in patient cohorts with chronic infection, even after viral loads diminished (109). Attempts to expand these cells *in vitro* were unsuccessful despite verification of their presence early in infection. In addition to the reduction of T_H_1 associated cytokine production, many studies have also identified altered cytokine expression by CD4^+^ T cells characterized by elevated IL-10 and IL-21 expression (107, 110–112). CD4^+^ T cells can also develop inhibitory receptor expression patterns associated with T cell exhaustion by expressing CTLA-4, which was observed in LCMV, HIV, and HBV chronic infections; PD-1; CD160; and BTLA (108, 112–115). CD4^+^ T cells are known to have high levels of PD-1 during chronic HIV infection and in one study this was observed to correlate with viral load (116). However, despite high levels of PD-1 expression, these cells retained the ability to produce IFN-γ (117).

Models of persistent parasite infection using *T. gondii* suggest that CD4^+^ T cells also become exhausted, with overlapping features of Blimp-1 expression, decreased expression of co-stimulatory molecules including OX40, ICOS, and 41BB, and increased inhibitory receptor expression such as 2B4; further, a reduction in cytokine expression was observed (118). These comparisons were made based upon the graded levels of PD-1, with cells that express greater PD-1 considered to be a more exhausted phenotype (118). Persistent antigenic exposure appears to be the main driver of this phenotype as suggested by infection models with *Mycobacterium tuberculosis*, which demonstrate that the temporal availability of antigen affects cytokine expression and magnitude of the CD4^+^ T cell response (119). Cells with limited exposure to antigens developed into cells that would be considered stereotypical memory cells, while continuously stimulated CD4^+^ T cells have a functionally altered phenotype. Still other studies using mouse models of malaria infection showed that malaria-specific CD4^+^ T cells exposed to chronic *Plasmodium* spp. infection had reduced cytokine production in comparison to those cells first deprived of antigen, then subsequently re-exposed in infected hosts (120, 121). Other studies have suggested exhaustion in the context of *P. chabaudi* infection, based on reduced cytokine production capacity and proliferation comparing early times points and later time points post infection (122). In humans, more evidence is needed to support this claim in *Plasmodium* infections as few studies have been performed and phenotypic analysis of CD4^+^ T cells only suggests exhaustion by inhibitory receptor expression (123). Disregarding whether CD4^+^ T cells are admittedly exhausted or dysfunctional, many studies in both mouse and human in multiple different chronic infections support the premise that blockade therapy of PD-1, PD-L1, or CTLA-4 augments CD4^+^ T cell cytokine production or proliferation (113, 115).

T_H_1 CD4^+^ T cells are commonly generated as a result of both acute and persistent viral infection and can be critical for CD8^+^ T cell function; but in the case of persistent infections, this population can be lost over time (83). For example in HIV patients, over long term treatment there is a discernable decrease in Gag293-tetramer specific CD4^+^ T_H_1 cells whereas CMV-specific CD4^+^ T_H_1 cells in the same patients remain unchanged (124). An important role for T_H_1 cells in chronic infection however is highlighted by the finding that in HIV controllers, individuals who control HIV replication without antiretroviral therapy, a polyfunctional T_H_1 CD4^+^ T cell population is maintained. As a note of caution, HIV-specific CD4^+^ T cells are often compared to CMV-specific CD4^+^ T cells in terms of function and phenotype, despite major differences in the course of infection and viral replication kinetics. Antigen availability and viral load may also play an important role in T cell differentiation. Therefore, the characteristics of different pathogens and antigen exposure clearly play a role in T cell differentiation and function (119). A similar phenomenon was described in HCV patients, in which patients who responded to interferon-α treatment had better maintenance of a polyfunctional HCV-specific T_H_1 CD4^+^ T cell population over non-responders (125). It was shown that CD4^+^ differentiation during chronic or prolonged antigenic stimulation in the context of infection skews CD4^+^ T cells toward a T follicular helper (T_FH_) cell lineage which may account for the loss of T_H_1 cell cytokine production. Recent studies have therefore focused on the contribution of the T_FH_ cell population during chronic infection (126). Indeed, T_H_1 and T_FH_ cells are generated early during infection with LCMV Cl-13 but the T_H_1 population is not maintained (127). This enrichment of T_FH_ cells during chronic phases was also observed in SIV (simian immunodeficiency virus) models with rhesus macaques, as it was noted that chronically infected rhesus macaques had increased T_FH_ cells and this correlated with elevated IL-6 levels, the cytokine known to induce T_FH_ differentiation (128). Others had noticed a similar trend but suggested that CD4^+^ T cell differentiation was being redirected toward a T_FH_ phenotype (129). T_FH_ cell skewing is not only observable in viral infections as patients with chronic parasitic infection, Schistosoma, show increased numbers of T cells with a T_FH_ phenotype that correlated with parasite-specific antibody levels (130). The development of a late T_FH_ phenotype was also present in the chronic phase of *Leishmania infantum* infection in rhesus macaques where there was an elevation in transcripts of *Bcl6, Cxcr5*, and *Il21*, all molecules associated with a T_FH_ response (131).

T helper cell differentiation is also observed during SIV infection, however new designations of “type 1 induced T_FH_ cells” have been adopted to account for those T_FH_ cells which have features of T_H_1 cells, including expression of CXCR3 and IFN-γ, but are more phenotypically T_FH_ by transcription factor and surface cell marker expression (132). An interesting study in SIV-infected rhesus macaques probed the question from the opposite perspective and sought to determine the kinetics of IL-21 expression during infection (133). IL-21 was produced by multiple T_H_ cell subsets, but predominantly T_H_1 cells and this early expression of IL-21 in T_H_1 cells negatively correlated with viral load, demonstrating the importance of a polyfunctional CD4^+^ T cell response in the early stages of a chronic infection (133). Variability in cytokine expression of CD4^+^ T cells suggests that the initial classification of CD4^+^ T cells into subsets based on cytokine production and transcription factor expression will likely need to be revisited in the context of chronic antigen stimulation in infections. What is unclear is whether persisting T_FH_ exhibit features of effector, memory, or exhausted T cells. As RNA-seq becomes more widely used as well as the ability to obtain transcriptomes of fewer cells using single-cell RNA-sequencing (scRNA-seq), the degree of heterogeneity in the CD4^+^ T cell population is becoming more apparent and will likely lead to new insights into CD4^+^ T cell responses to chronic antigen stimulation (112, 134).

At present, with the observation that there may be skewing of CD4^+^ T cell subsets during chronic or prolonged antigenic exposure, there are a few explanations as to the mechanism by which this occurs. Due to constant replication, studies with LCMV suggest that the exposure to type I IFN inhibits the *de novo* T_H_1 differentiation; this was first only surmised to be an indirect effect on CD4^+^ T cells as IFN receptor deficient CD4^+^ T cells did not augment the number of T_H_1 cells (127). Later experiments would support this claim, demonstrating that type I IFN induced IL-10 and PD-L1 on dendritic cells that would then suppress T_H_1 differentiation, and the subsequent loss of T_H_1 help would contribute to CD8^+^ T cell dysfunction (83). T_FH_ cell differentiation is likely driven by IL-6 that is produced later during the course of chronic LCMV infection (135). Recent studies in mice lacking the TCR scaffolding protein CD2AP, thus resulting in altered TCR signal strength, demonstrated increased T_FH_ generation and a concomitant increase in neutralizing antibody activity in LCMV which implies a role for TCR signaling in T_FH_ generation during infection (136).

Glucocorticoid-induced tumor necrosis factor related protein (GITR) is another molecule that was demonstrated to be important for CD4^+^ T cell differentiation during chronic LCMV infection (137). GITR-deficiency was shown to inhibit CD4^+^ T cells in the early T_H_1 production of IL-2 that is needed to support CD8^+^ T cell proliferation as well as the late T_FH_ cell response to promote humoral immunity through provision of B cell help. Thus, it remains possible that T_H_1 and T_FH_ are both generated during the initial infection and T_H_1 cells are not maintained during chronic infection. This differentiation toward a sustained T_FH_ cell presence during chronic infection appears to provide many benefits to the immune response. T_FH_ are named for their role in providing help to B cells and orchestrating the germinal center reaction (138). Importantly, resolution of chronic viral infection with LCMV is dependent on antibody production promoted by T_FH_ cells (139, 140). The importance of T_FH_ in HIV is also well-noted as the number of these circulating cells positively correlated with the presence of broadly neutralizing antibodies (141). During chronic or prolonged infections, many have observed the production of IL-21 by additional CD4^+^ T cell subsets including T_FH_ and T_H_17 cells (112, 142). Although typically associated with its importance in the germinal center reaction, in the context of chronic or prolonged infection, this cytokine has been shown to support CD8^+^ T cell function. Early studies in the LCMV chronic infection model noted the importance of CD4^+^ help to CD8^+^ T cells in the form of IL-21, however this appeared to come at a cost of reduced T_H_1 cytokine production in CD4^+^ T cells (143).

In the LCMV model, IL-21 signaling was linked to the induction of the transcription factor BATF in CD8^+^ T cells, which is important for maintenance of CD8^+^ T cell effector function (106). Similar evidence for IL-21 production preventing CD8^+^ T cell exhaustion during chronic infection was observed in a mouse model of parasitic infection using *T. gondii* (144). Beyond its role in the CD8^+^ T cell response, IL-21 deficiency was also observed to compromise the humoral arm in *T.gondii* infections, leaving mice more susceptible to toxoplasmic encephalitis (145). Lack of IL-21 signaling by global deletion of the IL-21 receptor (IL-21R) brought about increased inhibitory receptor expression on CD8^+^ T cells concomitant with greater parasite burden and reactivation (144). This susceptibility due to IL-21 insensitivity was also observed in a mouse model of tuberculosis (146, 147). When considering HIV in humans, small populations of IL-21-producing CD4^+^ T cells were present in the blood of patients with acute and chronic HIV and a greater frequency of HIV-specific CD8^+^ T cells expressed the IL-21R when compared to CMV-specific T cells (148). Combined with data suggesting that IL-21 ligation of IL-21R on HIV-specific CD8^+^ T cells enhanced effector molecule production, these findings support the role of CD4^+^ T cell derived IL-21 in providing necessary help to sustain CD8^+^ T cells during chronic infection (149). In studies of HIV/HCV co-infected individuals, these IL-21 producing CD4^+^ T cells were also associated with viral control, further supporting the role of this cytokine in antiviral immunity (150). Although attributed to T_H_17 cells, in SIV infection of rhesus macaques, IL-21 supported CD8^+^ T cell responses and prevented exhaustion (151).

Compared to CD8^+^ T cells, more information on CD4^+^ T cell differentiation during chronic infection is needed to accurately determine what effect chronic antigenic stimulation has on T helper cell differentiation and function. Whether T_FH_ or IL-21-producing CD4^+^ T cells that persist with time after chronic infection form “memory-like” cells has yet to be studied. Of note, this review does not discuss the implications chronic antigenic stimulation has on the development or differentiation of regulatory CD4^+^ T cells, or the levels of inhibitory receptor expression and suppressive cytokine production expressed by these cells. Many of the studies discussed, however, highlight the plasticity and heterogeneity present within the helper CD4^+^ T cell population as an adaptive immune cell that appears to be dynamically regulated by temporal and environmental dimensions. As noted above, future transcriptome studies utilizing scRNA-seq will enable further insight into the regulators that determine CD4^+^ T cell fate during chronic infection but also the profile of these cells. These studies can also help answer the question of whether the different antigen specific CD4+ T cell subpopulations are selectively lost as a result of chronic infection or their differentiation is skewed toward alternative differentiation lineages as the “memory-like” compartment develops. More recent studies have already hinted at the limitations of staining for a few markers and the possibility that CD4^+^ populations are much more polyfunctional than previously anticipated (133). This polyfunctionality of CD4^+^ T cell subsets, as demonstrated the ability of different cells to contribute to both humoral and cellular immunity (e.g., T_H_1 and T_FH_), highlights the importance of the different CD4^+^ T cell compartments and warrants further research to understand the dynamics and differentiation during chronic infections, and whether “memory-like” CD4^+^ T cells contribute to the sustained responses to chronic infections.

## T Cell Responses to Cancer and Cancer-Associated Antigens

Although our knowledge of effector, memory, and exhausted T cell differentiation largely comes from studies using virus and other infection models, it is crucial to better understand the extent of memory T cell formation in response to tumors as this can instruct the development of novel cancer treatments and aid in the development of vaccine strategies against cancer, particularly as recent studies have similarities between T cell subsets derived from chronic infection and tumors (152). From studies in both mice and humans, it is becoming more appreciated that the efficacy of anti-tumor responses is enhanced by the generation of both CD4^+^ and CD8^+^ “memory-like” T cell compartments.

### CD8^+^ Memory T Cell Development in Tumors

The priming of tumor-specific CD8^+^ T cells occurs in the lymph nodes by DCs that take up and cross-present neoantigens from the tumor, and activated tumor-specific T cells migrate into tumors guided by cytokine gradients (153, 154). Highly cytotoxic tumor-specific effector CD8^+^ T cells are a fundamental component of protective tumor infiltrating lymphocytes (TIL), and strongly correlate with patient survival (155, 156). After tumorigenesis, as with chronic infections, tumor-specific CD8^+^ T cells can become progressively dysfunctional and further persistence of the tumor can ultimately lead to the establishment of a permanent state of exhaustion (157, 158). At this stage, exhaustion cannot be reversed by anti-PD-1 therapy due to epigenetic modifications that prevent transcription of genes associated with effector function (158). As found with chronic virus infections, a major defining characteristic of T_EX_ in tumors is the increased expression and co-expression of multiple inhibitory receptors that include PD-1, Tim3, LAG3, CD160, and TIGIT, the absence of the transcription factor Tcf-1 with high expression of TOX, and progressive reduction in effector functions that are linked in part to dysregulated metabolism (77, 159). The critical role of TOX in the development of CD8^+^ T_EX_ in both chronic virus infections and cancer has only recently been described, with several studies identifying the necessity for TOX in T_EX_ development. These studies show a role for TOX in regulating chromatin accessibility/epigenetic modifications associated with T_EX_, and its expression is driven by NFAT and chronic TCR stimulation (70–73). However, dysfunctional tumor-specific CD8^+^ T cells can display two different chromatin states: a plastic and fixed dysfunctional state (160). Those cells within the fixed dysfunctional chromatin state are resistant to reprogramming and express high levels of CD38 and CD101, whereas PD-1^+^ TIL lacking CD38 and CD101 can undergo reprogramming to develop into effector cells (160).

Alterations in surface marker expression are determined by the transcriptional profiles of tumor-specific CD8^+^ T cells that define the differentiation states of the cells including “memory-like” CD8^+^ T cell compartments. Transcriptome analysis of tumor-specific CD8^+^ T cells from non-small cell lung carcinoma (NSCLC) and melanoma patients has identified the altered expression patterns of several transcription factors known to be major regulators of effector and memory CD8^+^ T cell differentiation, including Blimp1, Id2, T-bet, and Eomes (65, 161, 162). Phenotypically, in addition to expression of various inhibitory receptors, PD-1^hi^ CD44^int^ Eomes^hi^ CD8^+^ T cells exhibited a terminal T_EX_ cell phenotype, whereas PD-1^low^ CD44^hi^ Eomes^lo^ T-bet^hi^ CD8^+^ T cells could form effector cells. Terminal T_EX_ cells are characterized by high expression of Eomes and decreased levels of T-bet. First defined in chronic LCMV infection, PD-1^+^CXCR5^+^Tim3^−^ CD8^+^ T cells in the lymphoid organs were found to be responsive to PD-1 blockade therapy and express the transcription factor Tcf-1 while sharing a common gene signature with CD8^+^ memory precursors and were subsequently denoted as T_SC_ (49). Similar to virus-specific T_SC_, intratumoral melanoma tumor-antigen-specific Tcf-1^+^PD-1^+^CD8^+^ T cells exhibit stem-like properties that include self-renewal and proliferation and expanded in response to checkpoint blockade were found to have characteristics of both T_EX_ and T_SC_ (163). In melanoma patients, the Tcf-1^+^PD-1^+^CD8^+^ T cell population increased in response to anti-CTLA-4 and/or anti-PD-1 treatment and there is the potential that detection of this population can predict patient survival. CX3CR1 expression is also associated with increased responsiveness to PD-1 checkpoint blockade therapy, as increased expression of CX3CR1 on CD11a^+^CD8^+^ T cells in NSCLC patients strongly correlated with a positive clinical response to treatment (164).

Because of the recognized heterogeneity of CD8^+^ T cells within tumors, the use of single-cell analysis techniques is yielding important new insights into the unique properties of tumor-specific T cells. A recent study from Sade-Feldman et al. used scRNA-seq to address whether patterns in the tumor transcriptome could predict patient responses to checkpoint blockade therapy (162). In comparing the transcriptomes of tumors from 48 melanoma patients, their study highlighted the heterogeneity of the CD8^+^ T cell compartment and identified a strong correlation between the expression of Tcf-1 in CD8^+^ T cells and clinical responses to checkpoint blockade (162). Although they did not detect an association with CXCR5 expression and T cell responsiveness in their patient population as was found in previous studies, they showed that expression of CD39 was indicative of CD8^+^ T_EX_ cells. Several recent studies have also shown that TILs are a highly diverse T cell pool. Li et al. found that CD8^+^ TILs from melanoma patients form a gradient of dysfunction as indicated by transcription factor and inhibitory receptor expression (165). Furthermore, dysfunctional CD8^+^ T cells maintained the ability to clonally expand in the early phase of tumor progression (165). In a study of hepatocellular carcinoma (HCC), scRNA-seq analysis not only highlighted an enrichment of CD8^+^ T_EX_ in HCC, but also identified a CX3CR1 cluster of effector “memory-like” CD8^+^ T cells, drawing parallels to the findings in NSCLC (166). At this junction, it does appear that these cells, which are only found in the context of chronic antigen stimulation, can be considered to be memory cells despite some overlap in gene signatures with T_EX_.

In both humans and mice, there is evidence supporting the development of tumor-specific “memory-like” CD8^+^ T cells, which may be favored at the early stages of tumor growth when the extent of inflammation and levels of antigen exposure are reduced compared to later stages of cancer progression. In melanoma patients that received adoptive T cell therapy, it was shown that the infused CD8^+^ T cells developed a T_CM_ phenotype *in vivo* (167). Further, in some patients with colorectal cancer, T_EM_- and T_CM_-like populations have been identified, demonstrating the possibility that memory CD8^+^ T cells may naturally develop in response to cancer antigens. In one study, CD8^+^CD45RO^+^CCR7^−^CD28^+^CD27^+^ effector memory phenotype T cells were detected within colorectal tumor resections and were associated with increased survival in patients and noted a positive correlation between the infiltration of “memory-like” CD8^+^ T cells and patient survival (168). In particular, high levels of “memory-like” CD45RO^+^ cells within the tumor strongly correlated with the absence of early metastatic disease. In breast cancer patients, it has been shown that the ratio of the “memory” T cell compartment (CD45RO^+^) compared to naïve T cells in the bone marrow was significantly increased for both CD4^+^ and CD8^+^ T cells in patients compared to healthy controls, with the greatest increase in memory phenotype CD4^+^ T cells in bone marrow of patients where disseminated tumor cells were detected. Although the significance of these findings is unclear, this study found that despite an initial increase in HLA-A2/Her-2/neu_369−377_ tetramer-binding tumor specific “memory-like” T cells in the bone marrow, as the tumor advanced to later stages, this population ultimately decreased (169). This could potentially indicate a role for antigen load in the deletion or distribution of “memory-like” tumor-specific T cells. From mouse studies, it is thought that T_CM_ may be more protective and effective against cancer compared to T_EM_, in part due to their high levels of IL-2 production and capacity for proliferation (170). One study found that, on a per-cell basis, *in vitro*-generated tumor-specific T_CM_-like CD8^+^ T cells were able to mount a strong recall response to tumors greater than that of their T_EM_-like cultured counterparts and were capable of eradicating established tumors when combined with both exogenous IL-2 and a cancer-antigen vaccination strategy (170).

Vaccination strategies have also been employed to promote the development of tumor-specific memory CD8^+^ T cells, recognizing the importance of these T cells in achieving long-term tumor control. In one very promising study in mice, vaccination was applied after tumor excision. Following excision of primary B16 melanoma tumors, mice were vaccinated with optTRP1_455_ peptide and also given TGF-β blockade to reverse the tumor and regulatory CD4^+^ (T_reg_) cell TGF-β-mediated suppression of CD8^+^ T cells (171). Strikingly, upon re-challenge with B16 tumors, mice that had received both treatments showed increased protection with 50% of mice failing to develop tumors. This was attributed to the development of a protective CD8^+^ T cell population characterized by the stronger prevalence of tumor-infiltrating CD8^+^ T cells with a memory precursor phenotype. Another study aimed at exploring the impact of T_regs_ on limiting the development of effective CD8^+^ T cell responses to B16 melanoma. This study found that prophylactic depletion of T_regs_ by anti-CD25 treatment prior to primary tumor engraftment and followed by primary tumor resection resulted in protection of 80% of mice against secondary tumor growth re-challenge (172). Further, deletion of the bulk CD4^+^ T cell population allowed for long-lived antigen-specific CD8^+^ T cells in secondary lymphoid organs and were protective after primary tumor resection against both localized and systemic secondary tumor challenges. While these studies demonstrate the potential for “memory-like” CD8^+^ T cell formation in response to tumors, it is unclear how long these “memory-like” populations persist in patients and their efficacy in protecting against relapse. It is equally important to recognize that these populations may only arise in cancers that are more localized (e.g., breast cancer, melanoma) rather than systemic (e.g., leukemia or lymphoma) and that the rate of disease progression may play a major role in determining if “memory-like” CD8^+^ T cells will form.

Recently, there has been great interest in the T_RM_ compartment in cancer due to their function in local protection against repeat infections (173). Previous studies have shown that human lung tumor-infiltrating CD8^+^ T cells express high levels of CD103 and CD69, and low levels of CD62L and CCR7, suggestive of T_RM_ which retain characteristics of activated cells and induce rapid and effective responses against disease (174). In the tumor microenvironment, abundant TGF-β and T cell receptor signaling through the tumor antigen/MHC class I (MHC-I) complex has been shown to induce the formation of tumor-specific CD8^+^CD103^+^ T cells (175). TGF-β signaling triggers CD103 expression on T cells, and enhances the lytic function of anti-tumor CD8^+^ T cells (176). T_RM_ cells in human lung cancers express high levels of granzyme B, perforin, CD107a, and IFN-γ (177). Further, CD103 interactions with E-cadherin induces CCR5-mediated recruitment of CD8^+^ T cells into tumor as well as polarization and exocytosis of cytolytic granules, ultimately leading to tumor cell lysis (178). Tumor-infiltrating cells with a T_RM_ phenotype from advanced melanoma and lung cancer patients express higher inhibitory receptors such as PD-1, Tim3, and LAG3, which opens up the possibility that checkpoint blockade might promote the greater anti-tumor immunity by T_RM_ cells (177, 179, 180). Studies in mice have provided encouraging evidence for the ability of PD-1 blockade therapy to promote the infiltration of T_RM_-like (CD69^+^CD103^+/−^) CD8^+^ OT-I T cells generated from transferred vaccination-derived T_CM_ (CD44^+^CD62L^+^) into both B16-OVA and MC38-OVA (181). In both model systems, the addition of PD-1 blockade resulted in better tumor control and increased numbers of T_RM_-like donor OT-I cells per gram of tumor. As the prevalence of checkpoint blockade therapy in patients grows, it will be important to evaluate how these therapies contribute to the development of “memory-like” CD8^+^ T cells in patients that are in remission. Losing the potentially beneficial contribution of TGF-β to T_RM_ formation in the tumor microenvironment must therefore be considered when thinking about therapeutic TGF-β to limit CD8^+^ T cell inhibition.

At the molecular level, T_RM_ cells do not express Eomes and Tcf-1, which are expressed by other memory T cell subsets [Table 1, (182, 183)]. Absence of Eomes expression is required for CD103 induction and low expression of T-bet is necessary for expression of CD122 and maintaining IL-15 responsiveness by T_RM_ cells (175). On the other hand, expression of the transcription factors Hobit (homolog of Blimp1 in T cells) and Blimp1 promote the retention of T_RM_ cells in multiple organs and suppress genes related to egress from tissues (86). Runx3 is required to form T_RM_ cells in various tissues and tumors (62), and the transcription factors BATF (which is essential in the differentiation of effector T cells) and NAB1 (which is proposed to prevent apoptosis of TILs) are also upregulated in T_RM_ cells in tumors (177). Although the function of T_RM_ cells in anti-tumor immunity has not yet been fully addressed, accumulating data indicates that the cells can have a crucial role in anti-tumor responses (30). Malik et al. showed that skin-resident T_RM_ induced by vitiligo have a CD103^+^CD69^+^ phenotype and are beneficial in protecting against melanoma (126). In untreated lung cancer patients, the density of CD103^+^ T_RM_ cells among tumor-infiltrating CD8^+^ T cells shows a high potential as a prognostic markers for increased patient survival (177). Similarly, CD103^+^ TILs from high-grade serous ovarian cancer (HGSC) correlate with better patient survival (184).

Taken together, the studies of CD8^+^ T cells in anti-tumor responses support the possibility of generating *bona fide* tumor-specific memory particularly in the context of localized tumors and as a consequence of vaccination strategies with tumor-specific epitopes that can be generated by cancers with frequent mutations. Moreover, with adoptive cell therapies such as those based on TILs, it may ultimately be possible to preselect memory cells to develop infusion products that can become established as memory cells and thereafter maintained to protect against re-emergence of tumors such as observed with the persistence of the chimeric antigen receptor (CAR) T cell therapies (185).

### CD4^+^ T Cell Memory Development in Tumors

While the main focus of basic and clinical research has been on improving CD8^+^ T cell-mediated eradication of tumor cells, the role of CD4^+^ T cells in tumor immunotherapy is much less developed. Moreover, evidence for the involvement of CD4^+^ T cells in tumor eradication extends beyond the canonical function of helper T cells and their ability to promote CD8^+^ T cell and B cell responses. These include direct effects on tumor cells by cytokines produced by CD4^+^ T cells such as IFN-γ, TNF-α, and IL-2, modulation of DCs and other antigen presenting cells in the tumor microenvironment as well as direct killing of tumor cells by cytolytic CD4^+^ cells. As such, promoting CD4^+^ responses to tumors and the generation of CD4^+^ T cell memory are crucial to developing an effective anti-tumor immune response.

It has been known for some time that MHC class II-restricted (MHC-II) tumor antigens were capable of initiating CD4^+^ T cell responses critical for maintenance of anti-tumor immunity (186). More recently, MHC II-restricted neoantigens were found to possibly be more effective targets for cancer immunotherapy (187). Using these neoantigens in tumor targeted vaccine-based strategies is thus an important consideration for promoting memory development. In certain tumors such as breast cancer, the presence of memory phenotype T cells are a prognostic indicator for anti-tumor responses, with an increase in T_CM_-like and decrease in T_EM_-like CD4^+^ cells in the lymph nodes of patients progressing from stage I to stage III disease (188). Similarly, an increase in intratumoral CD4^+^ T_EM_ in colorectal tumors correlated with disease-free and survival rates in patients (155, 189). In the case of immune checkpoint blockade therapy, it was recently shown that an increase in a subset of central “memory-like” (CD27^+^Fas^−^CD45RA^−^CCR7^+^) CD4^+^ T cells in patients with malignant melanoma could be used as a predictor of clinical response to PD-1 blockade therapy (190, 191). In fact, CD4^+^ T cell memory could be induced by tri-specific antibody treatment targeting immune checkpoint inhibitors to the tumor and activating tumor-specific both CD4^+^ and CD8^+^ T cells simultaneously, with the greatest effect observed in the CD4^+^ T_EM_ and T_CM_ compartments in mice (192). Thus, there is great therapeutic potential in harnessing the power of memory CD4^+^ T cells to promote the most effective anti-tumor immune responses.

Although cytotoxic CD8^+^ T cells have been the focus of eliciting an anti-tumor response, it is clear that this response benefits from CD4^+^ T cell help and it has been shown that cross-priming of CD8^+^ T cells by DCs requires CD4^+^ T cell help for effective cytotoxic CD8^+^ T cell responses (193–195). DCs involved in the initiation of the anti-tumor T cell response also benefit from CD4^+^ T cell help, as CD40/CD40L interaction with CD4^+^ T cells is required to fully activate DCs that can subsequently generate CD8^+^ T_EFF_ and long-lasting CD8^+^ T cell memory (196). Further, it has been shown that T_H_1 cells can induce cytotoxic DCs that can kill tumor cells (197). Conversely, inhibition MHC-II antigen presentation by DCs to CD4^+^ T cells also promotes the development of anergic anti-tumor CD8^+^ T cells (198). PD-1^+^ tumor-specific CD8^+^ T cells are found in the blood of melanoma patients, indicating that priming of these T cells has occurred, although these cells are largely dysfunctional and resemble T_EX_ cells that develop during chronic infections (158, 199). Interestingly, these T_EX_ cells also are very similar to T cells which have not received CD4^+^ T cell help, suggesting that the tumor specific CD8^+^ T cells identified following initial priming by DCs did not see CD4^+^ T cell help at that time. These cytotoxic CD8^+^ T cells have been shown to be excluded from the tumor microenvironment in part due to TGF-β signaling (200). This exclusion is associated with poor clinical outcome as well as poor response to immune checkpoint blockade therapy (155, 201). Moreover, CD4^+^ help during priming can provide the signals needed to promote invasiveness of cytotoxic CD8^+^ T cells (202, 203). In addition, polyclonal CD4^+^ T cells from MHC-II-negative ovarian cancer tumor-bearing mice were able to secrete CCL5 and recruit CCR5^+^ DCs to the tumor (204). This was also shown to be important to optimize CD4^+^ T cell help to cytotoxic CD8^+^ cells as CCR5 ligands can improve the anti-tumor response (205, 206). Although some tumor cells do not express MHC-II, it has previously been shown that CD4^+^ T cells can still mediate rejection of these MHC-II-deficient tumors through indirect mechanisms and there is also evidence for the development of a CD4^+^ T cell anti-tumor memory compartment in breast cancer patients and in the B16 melanoma mouse model (198, 207–209). In breast cancer patients, analysis of bone marrow detected both T_CM_ and T_EM_ phenotype CD4^+^ T cells, and the adoptive transfer of these cells into NOD scid mice with patient tumor transplants showed infiltration of these cells into the tumors (210). This suggests that CD4^+^ T cell help promotes CTL responses through the recruitment of functional CD8^+^ T cells primed by DCs and capable of migrating into the tumor. In a Her2-positive breast cancer model in mice, one study found that bulk “memory” CD4^+^ T cells from viral immune-oncotherapy cured tumor-bearing mice proliferated upon either *in vivo* or *in vitro* challenge (211). In B16 melanoma, administration of DCs loaded with apoptotic B16 cells to mice promoted the development of a long-lived functional anti-tumor CD4^+^ T cell compartment that produced IFN-γ upon stimulation. Importantly, this compartment was highly protective as mice subsequently challenged with B16 tumors were protected unless CD4^+^ (or CD8^+^) T cells were depleted prior to tumor challenge (212). Taken together, these studies demonstrate that generating the formation of a long-lived, functional “memory-like” CD4^+^ T cell compartment can provide anti-tumor immunity. In addition, the long-lived and highly proliferative population resembling T_SC_ cells can be generated *in vitro* by activating CD4^+^ T cells by co-culture with stromal cells expressing Notch ligands (213). Importantly, these cells can expand and develop into tumor-specific effector cells after restimulation, a promising prospect for adoptive cell immunotherapy.

Thus far, the development of cancer vaccines solely focusing on CD8^+^ T cell epitopes has not been particularly successful without considering CD4^+^ T cell help (214, 215). Immune adjuvant therapy, the administration of an immune stimulant in connection with treatment, has been found to be beneficial in generating anti-tumor immunity by promoting T cell memory (216, 217). As an example, in breast cancer patients, peptide vaccination using the E75 peptide in combination with GM-CSF in breast cancer patients was able to activate both naïve CD4^+^ T cells as well as memory-phenotype CD4^+^ T cells specific for the tumor. Sustained anti-tumor CD4^+^ T cell “memory-like” formation was also shown in a vaccine trial of prostate cancer patients utilizing the AE37 vaccine and the DR11/AE37 tetramer to identify AE37 specific T cells. AE37 specific CD4^+^ T cells were detected up to 4 years following vaccination, and retained responsiveness as shown by peptide stimulation (218). Work by Bergman et al. has shown the effectiveness of generating potent anti-tumor CD4^+^ memory response (211, 219). These studies utilized viral oncolytic immunotherapy to prime T cell responses that were otherwise suppressed by chemotherapy-based regimens. Memory recall capability was shown by adoptive cell therapy and while transferred CD8^+^ T cells were poor in controlling tumor growth, transfer of memory CD4^+^ T cells was capable of resolving established tumors, albeit when injected in high numbers. Therefore, any consideration of adoptive immune cell therapy or cancer vaccines should include promoting the development of antigen-specific memory CD4^+^ T cells.

Even though providing help is a major role for CD4^+^ T cells in anti-tumor immune responses, CD4^+^ T cells can contribute directly to regulation of the tumor microenvironment and to killing of cancer cells (220, 221). It was suggested that CD4^+^ cells kill tumor cells through a mechanism that did not involve Fas/FasL or TNF-α, but was dependent on the TNF-α related apoptosis inducing ligand (TRAIL) (222). T_H_1 CD4^+^ T cell responses can support anti-tumor immunity, in part due to the direct impact IFN-γ has on tumor cells (194). One study described T_EM_ CD4^+^ T cells that were capable of tumor elimination and this was dependent on IFN-γ (223). Strikingly, tumor reactive cytotoxic CD4^+^ T cells could be induced following checkpoint blockade therapy (224). These CD4^+^ T cells expressed Eomes but not T-bet, secreted IFN-γ, expressed granzyme B and perforin, and were capable of lysing autologous tumor cells (224). Similarly, it was shown that OX40 engagement induced both cytotoxic and memory CD4^+^ T cells characterized by Eomes expression (221). These cells were capable of controlling tumors in mice and lysing human tumor cells *in vitro*. Thus, independent of their function in providing help, CD4^+^ T cells can be generated that can directly target cancer cells for elimination.

Taken together, these studies in both humans and mice identify not only the potential for memory anti-tumor CD4^+^ and CD8^+^ T cell development, but also highlight their strong anti-tumor potential. Moreover, developing new strategies aimed at generating optimal CD4^+^ T cell responses and memory in the context of chronic antigen exposure may offer treatments for cancers that are resistant to current immunotherapies.

## Discussion

The many studies discussed within this review demonstrate the possibility of memory CD4^+^ and CD8^+^ T cell generation under conditions of chronic or persistent antigenic stimulation. Perhaps most importantly, they highlight the high degree of diversity and heterogeneity of long-lasting and persisting memory or “memory-like” T cells generated in patients. The importance of this diversity is shown by the reproducible formation of highly heterogeneous memory T cells within genetically identical mice and with TCR transgenic T cell models where the T cell repertoire is defined (225). Memory T cell diversity, in part, reflects an array of persisting antigen-experienced T cells that have progressed through various stages of differentiation in different contexts of antigen exposure in different tissues. Indeed, a process of tissue “imprinting” can govern the migration and maintenance of memory T cells in sites such as the gut associated tissues, skin, and lung. A major contribution to memory T cell fate determination is the antigen dose and extent of the inflammatory milieu, which can drive the development of terminal effectors that are lost during the contraction phase as antigen becomes cleared (226). Indeed, exposure of T cells to lower levels of antigen at this stage of a response can favor the generation of memory cells with the capacity for self-renewal (227). A robust immune response and rapid pathogen clearance by the T cell response favors greater generation of such memory T cells, and it is cells with similar properties (e.g., Tcf-1 expression) that can respond to immune checkpoint blockade in the settings of chronic antigen exposure (49, 228). These observations underscore the concept that antigen-experienced memory T cells that retain functional and protective capabilities are generated during chronic exposure to antigens but are unable to respond because of suppressive mechanisms in the local environment. Factors like impaired antigen-presentation, limited T cell activation in response to TCR signaling, and metabolic suppression that impair to differentiate into secondary effectors and elicit control of chronic infections and cancers also inhibit the generation of memory T cells. Although we have identified some of the parameters that distinguish subsets of memory T cells and are beginning to clinically exploit properties that promote their function, it is clear that identifying strategies that promote the development of memory in the context of chronic antigen-exposure will be crucial.

Many publications also highlight the perhaps long-standing misconception that CD4^+^ and CD8^+^ T cells follow similar differentiation pathways or develop similar characteristics as a result of chronic antigenic stimulation. This may very well be a result of differences in peptide-stimulation itself, as CD8^+^ T cells encounter peptide on nearly all nucleated cells in the context of MHC-I while CD4^+^ T cells are somewhat more protected from this constant bombardment of antigen by the more restricted expression of MHC-II. Indeed, many autologous T cell transfer strategies aim to expand T cells *ex vivo* and in turn provide them with a period of antigen deprivation whereby T cells can be rested from these debilitating environments. These models also highlight not only the effect that the degree of antigen exposure has on T cell development, but also introduce a temporal aspect. Dysfunction is favored by longer duration of exposure to persistent antigen-stimulating environments that decreases the likelihood of “rescuing” these cells from dysfunctional differentiation states. Limiting antigen or reducing the time of exposure may reveal key aspects to direct future avenues for restoring the proper differentiation pathway of T cells exposed to chronic antigenic stimulation.

Another concept not fully discussed within this review is that of memory inflation, or the temporal increase in a T cell population with a virus-specific (tetramer^+^) “effector-memory” phenotype (CCR7^low^CD62L^low^CD28^low^CD27^low^) and accumulation of these cells in many non-lymphoid tissues. First defined in mouse models of murine CMV (MCMV), memory inflation has also been observed humans following CMV infection, parvoviruses B19 and PARV4, chronic norovirus, extreme responses to EBV, and to adenovirus-based vaccinations (229). It is currently understood that antigenic persistence is a requirement for memory T cell inflation and is believed to be driven by sites of latent virus infection, as removal of the primary site of viral replication (e.g., the salivary glands) does not stop the phenomenon of memory inflation (230). An important distinction however between “classical” T_EX_ formed under persistent antigenic conditions such as with LCMV Cl13 or HIV infections compared to T cells generated via memory inflation is the retention of effector cytokine production and an overall lack of T_EX_ hallmark features such as co-expression of inhibitory receptors. The localization of inflationary memory T cells in non-lymphoid peripheral tissues is a hallmark they share with T_RM_; however, while T_RM_ are confined to the tissue in which they were generated, a high number of inflationary memory T cells can be found in circulation after MCMV and CMV infection (229). Transcriptional profiling of both inflationary T cells and T_RM_ identified some commonalities between the two T cell types (e.g., upregulation of chemokine receptors and T-bet), but also showed significant transcriptional diversity (e.g., upregulation of AP-1 family members in T_RM_, and IRF8 and EZH2 in inflationary T cells) (231). This distinction further highlights the high potential for diversity in T cell differentiation and stresses the importance of understanding how antigen availability and persistence can influence the development of functional memory or “memory-like” T cells compared to exhaustion.

Evaluation of the contribution of CD4^+^ T cells is often neglected as CD8^+^ T cells have a more direct role in cell elimination; however, CD4^+^ T cells are important for both cellular and humoral immunity. As cells supporting both arms of immunity, they warrant further study into the roles they play during persistent antigen exposure and what affect they could have on promoting memory T cell formation. A loss of CD4^+^ T cell help contributes to CD8^+^ T cell dysfunction in chronic viral infection, but the potential for CD4^+^ T cells to form memory during chronic infection remains unconfirmed. In addition, more studies into the direct effects of CD4^+^ T cells on the tumor microenvironment and their contribution to the killing of cancer cells are needed. An important question that remains unanswered is whether the presence of a memory CD4^+^ T cell pool limits the establishment of secondary/metastatic tumors by affecting the tumor microenvironment. And further, to what extent is the maintenance CD4^+^ T cell help required to sustain the cytotoxic effector functions of CD8^+^ T cells within tumors? Further studies on CD8^+^ T cells are also needed to define parameters that limit CD8^+^ T cells development into true memory compartments, to address how dysfunctional differentiation pathways can be skewed toward successful memory, and to identify possible interventions to establish functional memory. As sequencing techniques have become more robust and with the advent of methods that allow for RNA transcriptome analysis to be performed on smaller cell numbers, a large emphasis has been placed on understanding the molecular determinants of memory T cell formation. Perhaps the greatest aide in understanding the complex and heterogeneous memory T cell pool has been the development of scRNA-seq, as this allows for the first time the evaluation of transcription factor co-expression and relative expression levels on the single-cell level. It also raises the question as to how different subsets arise from the same inflammatory environment and antigenic stimuli.

Not fully discussed in this review are the important findings regarding changes in epigenetics and their contributing role in T cell differentiation and particularly dysfunction, as these have been extensively and recently reviewed elsewhere (77). It is clear that changes in DNA methylation and chromatin structure play an important role in both CD4^+^ and CD8^+^ T cell fate decisions, and studies aimed at deciphering patterns in epigenetic remodeling of T cells during chronic infection and cancer have provided key insight into the regulation of T cells that are effective in killing infected or malignant cells (232, 233). Future studies combining evaluation of memory and exhausted T cells that arise during chronic antigen stimulation at both the epigenetic and transcriptome level may provide key insight into targets for therapies that promote the formation of beneficial T cell responses.

Greater consideration is now being given to the influence of metabolism on T cell differentiation and memory T cell development, particularly under the conditions of chronic or persistent antigen. Several groups have now demonstrated the unique metabolic requirements of the different T cell subsets, such as the glycolytic switch that occurs upon TCR stimulation and the subsequent switch back to fatty acid oxidation (FAO) and oxidative phosphorylation (OXPHOS) by *bona fide* memory T cells (234, 235). In chronic virus infections, it has already been demonstrated that exhausted T cells develop altered metabolism compared to functional virus-specific T cells, specifically an increased reliance on glycolysis and inability to use oxidative phosphorylation when exhausted (236). This may be a crucial distinction, since in the case of cancer, tumor cells can outcompete T cells for glucose (237). More recently, it has been shown that checkpoint blockade therapy can affect T cell metabolism, as both PD-1 and CTLA-4 signaling have been shown to inhibit glycolysis and PD-1 signaling promotes FAO in T cells (238, 239). As glycolysis has previously been linked to the production of inflammatory cytokines by T cells (240), this is an important consideration when trying to reverse T cell exhaustion in patients and promote memory T cell development as memory T cells have unique metabolic requirements as previously stated. Further, we are only now beginning to understand how the tumor's metabolism can impact T cell metabolism beyond nutrient deprivation and competition for glucose. The highly hypoxic tumor microenvironment promotes HIF1α expression in TILs, which further promotes glycolysis and decreased reliance on OXPHOS by the T cells. In general, TILs demonstrate major alterations in metabolism including defects in mitochondrial biogenesis and oxidative function (237, 241). Work from Delgoffe demonstrates how the state of the tumor (e.g., oxidative metabolism) can influence T cell responses to checkpoint blockade therapy and provide a predictive indicator to anti-PD-1 therapy responsiveness (241). Although tumor heterogeneity is often discussed in the context of antigen availability and “hot vs. cold” in terms of the presence of TILs, we may be overlooking the metabolic complexity of different tumor microenvironments and this significant contribution to T cell responsiveness. Better understanding the metabolic requirements of a highly effective TIL response in cancer and concurrently how the tumor metabolic requirements can be altered to generate a favorable TIL response could lead to an important convergence of anti-cancer therapies with a two-pronged approach.

Taken together, the studies summarized in this review highlight the complexities that must be considered when discussing and evaluating alterations in T cell responses and particularly when comparing memory formation with acute infection to conditions of chronic antigen stimulation. We are rapidly gaining greater insight into the molecular regulators of T cell dysfunction, effector generation, and memory development at both the transcriptional and epigenetic levels. Addressing how T cells interact with their microenvironment and the role of subsequent metabolic changes in the context of these important findings will be key in unlocking new strategies aimed at improving patient responses to chronic infections as well as cancer.

## Author Contributions

All authors listed have made a substantial, direct and intellectual contribution to the work, and approved it for publication.

### Conflict of Interest Statement

The authors declare that the research was conducted in the absence of any commercial or financial relationships that could be construed as a potential conflict of interest.

## Abbreviations

T_N_: Naive T cell
T_EFF_: Effector T cell
T_EX_: Exhausted T cell
T_CM_: Central memory T cell
T_EM_: Effector memory T cell
T_RM_: Resident memory T cell
T_SC_: Stem-cell like T cell
T_EMRA_: Terminally differentiated effector memory T cell
OVA: Ovalbumin
Lm-OVA: *Listeria monocytogenes*-OVA
OT-I: Transgenic mouse CD8^+^ T cells that recognize OVA (SIINFEKL)
TCR: T cell receptor
IFN: Intereferon
IL: Interleukin
T-bet: T-box transcription factor TBX21
Eomes: Eomesodermin
HSV: Herpes simplex virus
RSV: Respiratory syncytial virus
Blimp-1: B lymphocyte-induced maturation protein-1
Bcl-6: B-cell lymphoma 6
Bcl-2: B-cell lymphoma 2
Foxo: Class O of forkhead box transcription factors
FRC: Fibroblastic reticular cells
PD-L1: Programmed death-ligand 1
PD-1: Programmed cell death protein 1
CTLA-4: Cytotoxic T-lymphocyte-associated protein 4
HIV-1: Human immunodeficiency virus 1
HCV: Hepatitis C virus
T_H_1: Type 1 helper cell
IFN-γ: Interferon gamma
TNF-α: Tumor necrosis factor alpha
LCMV: Lymphocytic choriomeningitis virus
EBV: Epstein-Barr virus
CMV: Cytomegalovirus
HLA-I, Human leukocyte antigen: class I
CD: Cluster of differentiation
TB: Tuberculosis
LTBI: Latent tuberculosis infection
Hobit: Homolog of Blimp-1 in T cells
Tcf-1: T-cell factor 1
KLRG1: Killer cell lectin like receptor G1
CX3CR1: C-X3-C motif chemokine receptor 1
LAG3: Lymphocyte activating 3
ICOS: Inducible T-cell costimulator
T_FH_: T follicular helper cells
T_17_: T-helper 17 cells
SIV: Simian immunodeficiency virus
TIM3: T-cell immunoglobulin and mucin-domain containing-3
TIGIT: T cell immunoreceptor with Ig and ITIM domains
NSCLC: Non-small-cell lung carcinoma
scRNA-seq: Single cell RNA-sequencing
HCC: Hepatocellular carcinoma
TGF-β: Transforming growth factor beta 1
DC: Dendritic cell
MHC-I: Major histocompatibility complex class I
MHC-II: Major histocompatibility complex class II
GM-CSF: Granulocyte-macrophages colony-stimulating factor
FAO: Fatty acid oxidation
OXPHOS: Oxidative phosphorylation.
